# Correlations between Physical Fitness and Body Composition among Boys Aged 14–18—Conclusions of a Case Study to Reverse the Worsening Secular Trend in Fitness among Urban Youth Due to Sedentary Lifestyles

**DOI:** 10.3390/ijerph19148765

**Published:** 2022-07-19

**Authors:** Anetta Müller, Zsuzsa Nagy, Sándor Kovács, Szilvia Szőke, Elena Bendíková, Gergely Ráthonyi, Kinga Ráthonyi-Ódor, György Szabados, Zoltán Gabnai, Éva Bácsné Bába

**Affiliations:** 1 Department of Sports Economics and Management, University of Debrecen, H-4032 Debrecen, Hungary; muller.anetta@econ.unideb.hu (A.M.); rathonyi.gergely@econ.unideb.hu (G.R.); rathonyi-odor.kinga@econ.unideb.hu (K.R.-Ó.); szabados.gyorgy@econ.unideb.hu (G.S.); bacsne.baba.eva@econ.unideb.hu (É.B.B.); 2 Doctoral School of Humanities, University of Debrecen, H-4032 Debrecen, Hungary; nagizsuzsa@gmail.com; 3 Department of Research Methodology and Statistics, University of Debrecen, H-4032 Debrecen, Hungary; kovacs.sandor@econ.unideb.hu (S.K.); szoke.szilvia@econ.unideb.hu (S.S.); 4 Department of Physical Education and Sport, Faculty of Education, Catholic University in Ružomberok, 034 01 Ružomberok, Slovakia; elena.bendikova@ku.sk; 5 Department of Business Economics and Business Development, University of Debrecen, H-4032 Debrecen, Hungary

**Keywords:** health status indicators, fitness profile, exercise test, two-block PLS, daily P.E.

## Abstract

A secular trend can be observed throughout the world with an increase in childhood obesity and a decrease in fitness. The research aimed to examine the results of tests measuring the conditional abilities of young boys aged 14–18 in fitness tests and their correlations with body composition indicators. That was supported by research that has been prepared in Hungary so far. This research focuses on the results of fitness tests conducted on 14- to 18-year-old boys, presented along with body composition data. The authors sought to describe the development of the fitness profiles of males at a Budapest secondary school participating in the research, based on the results of the Hungarian National Student Fitness Test (NETFIT^®^), and also how their physical characteristics affect the results of NETFIT^®^ tests in the sample measured. A total of 735 male high school students at a Budapest secondary school (14–18 years old) (mean ± SD, 16.05 ± 1.18 years) participated in the survey. The data were collected in the 2018/2019 academic year, and it was compared with the national data. The correlation between the performance indicators of the NETFIT^®^ tests and the physical characteristic indicators was analyzed using the two-block Partial Least Squares method. In the resulting groups, Kruskal–Wallis variance analysis was performed to investigate the differences in performance. In contrast, pairs of group differences were tested with the Mann–Whitney test. Boys with a short physique were at some advantage in trunk-lifts and push-ups, compared to taller boys. It was also obvious that being overweight is a hindrance regarding the PACER test or the standing broad jump. The handgrip in the left and right hand was mostly of similar strength or weakness. Tall-heavy children performed better in this test. The grip strength of tall-thin students was also strong, but not as strong as in the tall-heavy group. Reducing the percentage of body fat (PBF) and creating the optimal BMI index is important for the younger age group, as our results have clearly demonstrated that overweight is a hindrance in the PACER, VO_2_ max, standing broad jump, back-saver sit-and-reach, and push-up tests.

## 1. Introduction

Compared to previous generations, it can be stated that our lifestyles have become increasingly sedentary. Fang et al. (2021) draw attention to the fact that sedentary time is becoming a serious global problem, with adverse public health implications [1]. The population is spending more and more time in environments that limit physical activity and that require prolonged sitting [2]. The COVID-19 pandemic has exacerbated this trend, and many countries have become accustomed to a new normal [3]. The terms “social distancing” and “local protection” are now part of everyday vernacular and life [3,4,5]. Several factors presumably influence poor participation in physical activity. Environmental factors include not only traffic congestion, air pollution, a lack of parks or pedestrian walkways, and a lack of sports and leisure facilities [6], but also conditions and negative trends in the work, home, and school environment. On the one hand, television viewing, video viewing, and mobile phone use are positively correlated with increasingly sedentary lifestyles [7], while Dixit-Nandakumar (2021) [8] highlights the opportunities offered by information technology to promote healthy lifestyles. However, it is predicted that sedentary behavior will continue to increase against this socio-cultural background [9]. The increased sedentary time due to the changing environment is valid for adults, young people, and children. A significant amount of literature has been written on the assessment of aerobic and general fitness among children, as well as on the obtained results and the changes observed in this secular trend [10,11,12,13,14]. According to Tomkinson-Olds (2007) [10], the factors associated with the secular trend are caused by a combination of social, behavioral, physical, psychosocial, and physiological factors, which can be primarily explained by higher caloric intake rates and reduced energy consumption associated with better access to technology. Ferrari et al. (2015) [15] analyzed changes in physical fitness over a 30-year period, in relation to schoolchildren’s nutritional status and gender. In their results, while confirming the trend observed at the global level, they found that the decline in physical fitness was greater for schoolchildren of normal weight than for those who were overweight. As another related result, Ortega et al. (2011) [16] found higher physical fitness in boys, except for the flexibility test, and that boys’ physical fitness increased with age, while girls’ fitness levels were more stable across ages, based on a study conducted on a sample of 3428 adolescents in 10 European cities.

Some research papers considered socioeconomic background as the most influential factor on fitness, and they confirmed regional differences in fitness results within a given country. The level of fitness among children living in regions with a more favorable socioeconomic background was found to be higher compared to that of children living in socioeconomically disadvantaged regions [17,18,19,20,21,22].

Several international studies have been written on childhood obesity and the decrease in children’s fitness, which all confirm the prevalence of this secular trend worldwide [23,24]. With regard to research on the assessment of the applied fitness tests, the study conducted by Armstrong and Welsman (2007) [25] confirmed a negative impact of BMI increase on aerobic fitness achievement. Arboix-Alió et al. (2020) [26] also highlight an alarming deterioration in the physical fitness of adolescents compared to previous decades, especially among urban populations, with respect to the evolution of cardiorespiratory fitness (CRF) over time, analyzing 20 years. Tomkinson et al.’s (2019) research, covering 33 years showed, a modest reduction in CRF [27]. They also found that the decline was greater in boys than in girls, and similar in children and adolescents. The magnitude and direction of trends also differed between countries. Tremblay et al. (2011) examined the relationship between sedentary behavior and health indicators in children and youth. They concluded that a reduction in any type of sedentary time was associated with a lower health risk [28].

The same secular trend can be observed in Hungary [29], making it clear that body fat increases in children lead to a decrease in their motor achievement. Therefore, the Hungarian Sport XXI National Sports Strategy 2007–2020 made it a priority to introduce daily physical education (P.E.) lessons, in order to increase children’s sports activity and fitness, which also seemed justified, given the growing prevalence of obesity in children [30].

In the academic year of 2012/2013, daily P.E. lessons were introduced in the curriculum, which were accompanied by the concomitant introduction of the compulsory assessment of school children’s fitness and body composition profile (NETFIT^®^). The time that has passed since their introduction offers the opportunity to study the assessment results regarding the fitness levels of the students taking part in everyday P.E. lessons.

The goal of the research was to examine the results of tests measuring the conditional abilities of young boys aged 14–18 in fitness tests, and their correlations with body composition indicators. This research is considered important because the life expectancy of men in Hungary is lower than women, and their health status is less favorable, so the role of prevention is becoming more and more important. A key research question was what could be determined regarding the sample’s fitness and body composition indicators at this young age. With the results, we would like to make a recommendation to the school locally, that will help create a healthier and fitter society.

The current research focuses on the results of fitness tests conducted on 14 to 18-year-old boys, which are presented, along with body composition data.

## 2. Materials and Methods

### 2.1. Participants

The research was conducted in schools directed by the Budapest Center of Technical Vocational Training, where the fitness level and body composition of secondary school boys aged 14 to 18 (*n* = 739) were assessed. The assessed students were included in the study after random sampling. The sample was chosen for this high school in Budapest, where there are only male students, because the authors assessed the tests themselves. The survey was performed by Zsuzsa Nagy, who taught physical education at this secondary school, so we could conduct all the surveys ourselves, which also ensured that the tests were assessed similarly. NETFIT^®^ data are recorded by all physical teachers in a central system; so that it is not possible to access the assessments of others in order to protect personal rights. These data are managed by the Hungarian School Sport Federation (MDSZ). Thus, it was possible to conduct the measurement in this school with the authors’ own survey. Out of the 783 people attending the school, 739 people were surveyed. Those students who could not participate due to absence from illness or health problems did not take part in the measurement. The sample is not representative.

The mean age of the assessed students was 16.05 years (SD = 1.181). A total of 40 of the enrolled students were 14 years old, 243 students were aged 15, 189 of them were 16 years of age, 168 students were aged 17, and 99 of them were aged 18 or above. This assessment, which is compulsory in Hungarian public education, was conducted in conformity with the requirements stipulated in the relevant legal provisions (Section 80 of Act CXC of 2011 on National Public Education). Students had been informed in advance about their data being used in an anonymized form in the study, and their parents had been asked to give their informed consent and to sign the appropriate form.

### 2.2. Ethical Approval

All procedures performed in studies involving human participants were in accordance with the ethical standards of the national research committee, and with the 1964 Helsinki declaration and its later amendments or comparable ethical standards. The research was performed with the ethical approval of the Medical Research Council Scientific and Research Committee, Hungary (50365-1/2015/EKU(0403/15)).

### 2.3. Measurements

This research employed the tests of NETFIT^®^, and the assessment was conducted in line with the methods determined and published by the Hungarian School Sport Federation (More information on NETFIT® tests is available at: https://www.netfit.eu/public/pb_fittsegi_tesztek.php and https://www.mdsz.hu/hir-english/hungary-tests-with-netfit/ (accessed on 12 December 2021)).

The survey was conducted in May 2019 in the gym of the Budapest high school. The same physical education teacher (Zsuzsa Nagy) assessed the students in each class. The survey took place by class and over several days, depending on the number of students in the class (2–4 days). The same person always conducted the warm-up before the measured tests, ensuring that the muscle groups used by the measured test were prepared for the increased load, ensuring the avoidance of the risk of injury and accident. Warmed-up muscles are more efficient, and the major training exercises are easier to perform because the body is prepared for them. The Hungarian School Sport Federation (MDSZ) provided all schools with the measuring tools used during the tests, and all the information related to the assessment of the tests (NETFIT^®^ book and video) was provided to the P.E. teacher in a training course, so the measurements take place in a similar way. Then, it was possible to compare the results obtained with the authors’ own measurements to the national average values, since the MDSZ publishes some of them, so the national data no longer come from our own measurements. The NETFIT^®^ test application is conducted based on an official manual [31].

The NETFIT^®^ assessment comprises seven motoric tests that measure skills determining students’ fitness levels. The tests in endurance profiles measure aerobic capacity or endurance, and the strength (relative strength and endurance) of certain muscle groups, while the test in the flexibility profile measures joint mobility and flexibility. In this study, six of these tests were assessed and analyzed. The seventh test is the flexibility test, which was not included in the results, because the research goal was to examine the development of conditional abilities, and this test did not show any correlation with the body composition indicators. The six tests include:

- 20 m PACER test (measures basic endurance),
- Paced curl-up test (measures muscular strength and endurance, especially that of the abdominal muscles),
- Trunk-lift test (measures the strength and elasticity of the spinal erector muscles),
- Handgrip strength test (measures the maximum strength of the handgrip),
- Paced push-up test (measures in a complex way the muscular strength of the upper part of the trunk, including the shoulder girdle and the arms, the back, the abdomen, and the lateral abdominal muscles),
- Standing long jump test (measures the dynamic muscle strength of the legs).

VO_2_ max was estimated on the basis of the 20 m pacer test, with the help of the following formula [32]:

VO_2_ max = 5.619 + 0.353 × (number of completed shuttle runs) − 1.121 × (age) (1)

In addition, body mass index (BMI) and relative body fat (%) were also measured to determine students’ nutritional status and their rate of obesity. The calculation of the BMI index and the assignment of students into obesity categories was performed based on the BMI-for-age percentile data published in Cole and Lobstein (2012) [33].

Body Mass Index (BMI) and relative body fat content (%) (provided by the OMRON BF511 for bioimpedance measurement help) were also determined to measure the nutritional status of the pupils and the extent of obesity. The BMI index calculation and classification into the obesity category were based on the body mass index relative to the age percentile [33].

### 2.4. Statistical Analyses

For model calculations, figures and statistical analyses, version R 3.4.4 of R CORE TEAM was used, and version 1.0.136 of RStudio (2016) [34] was used for the graphical environment. In order to measure the reliability of the studied variables between age groups, the so-called Intraclass Correlation Coefficient (ICC) was calculated for body composition and for performance variables, respectively. A two-way mixed effect model with consistency was applied for multiple measurements [35]. To further reveal the correlation between body composition and the performance variables of NETFIT^®^ tests, a two-block PLS (partial least squares) analysis was used. For the t-test calculation, the online calculator was applied, which is available at: https://www.quantitativeskills.com/sisa/statistics/t-test.htm (accessed on 10 December 2021) [36].

The intraclass correlation coefficient was also calculated for the different test measures as the ratio of the between-group (age) variance and the total variance (between plus within-group (age) variances). Its value ranges between 0 and 1. A value closer to 1 indicates that the total variance is rather due to the between-group variance than the within-group variance (age has a relatively higher importance on the given test measure).

The two-block PLS analysis is a method similar to factor analysis; however, there are two groups with the given variables, and the method will create different factors in the two groups. At the same time, factors in the two groups are not created independently, since the methods aim to maximize the correlations between two factor groups (further referred to as block 1 and block 2). The mathematical description of the entire algorithm can be found in Rohlf and Corti (2000) [37], and can be run using the Morpho package of the software R 3.4.4. [34]. In the framework of this method, the correlation matrix of the examined variables is grouped in line with the two blocks, then the SVD (Singular Value Decomposition) technique known from factor analysis is used exclusively for the cross-correlation matrix (R12) seen between the variables of the two blocks by using the following formula:

R12 = F1D(F2)t(2)

where matrix F1 and matrix F2 are the factor loading matrixes for the variables in block 1 and block 2 in all dimensions, and matrix D has the singular values in relation to factors, which can be used to provide the amount of information discussed concerning the variables in the two sets.

To determine the differences between NETFIT^®^ performance variables found in the groups set up by using the two-block PLS method [37], Kruskal–Wallis variance analysis [38] was used, while pairwise differences were detected using the Mann–Whitney test [38]. A significance level of 5% was used to determine statistically significant changes in statistical tests. Non-parametric statistical tests were also necessary, as the Kolmogorov–Smirnov test analysis [39] failed to show a normal distribution for most of the performance variables of NETFIT^®^. When comparing the results of the NETFIT^®^ test to the average national results, a parametric *t*-test [40] was used, since the original data were not available, and only mean values and standard deviation could be relied on. The Kruskal–Wallis test, the Mann–Whitney test, and the Kolmogorov–Smirnov test were conducted with SPSS Version 25.0. [41].

## 3. Results

### Comparison of the Data Obtained in the School against the National Data

National data were available for age groups. The data obtained in the school against the national data were compared using a t-test with the following variables: VO_2_ max, trunk-lift, paced push-up, handgrip strength, and standing long jump.

In the 14–18 age group, the *t*-test showed no difference between this study’s results and the corresponding national age group data on trunk-lift. The same could be observed among 14 and 18-year-old students regarding the result of VO_2_ max. The results that 14-year-old students achieved in paced curl-ups were also in line with the relevant national data. Regarding handgrip strength, the results for the 15, 16, and 18+ age groups were in harmony with the national data. In terms of the standing long jump, the results of the 14-year-old and 18+ students were similar to the national ones.

This research revealed that students in the study sample from Budapest had higher results in the paced push-ups in all the age groups compared to the national data, and this difference was quite significant. In the PACER test, which provided the VO_2_ max results, the 15, 16, and 17-year-old students achieved lower scores than the national data. In the paced curl-up test, the boys from Budapest had higher scores than the mean national results in the 15, 16, 17, and 18+ age groups. The handgrip strength of the 14-year-old boys came with higher scores, while the national mean proved to be higher among those aged 17. In the case of the standing long jump test, the results of 15-, 16-, and 17-year-old boys in the national sample provided higher mean values.

Table 1 provides a summary of the performance variables showing significant differences in the *t*-test.

VO_2_ max was estimated based on a 20 m PACER test with the following formula [32]:

VO_2_ max = 5.619 + 0.353 × (number of completed runs) − 1.121 × (age). (3)

The correlation was examined between the body composition and the fitness results of the assessed secondary school boys in the study sample in two ways. First, the ICC was calculated. Body composition values for the same age group can be considered as highly similar, as the ICC was 0.548 (95% confidence interval: 0.488–0.602; *p* < 0.001). The ICC was 0.338 (95% confidence interval: 0.256–0.413; *p* < 0.001) for fitness results showing a good similarity between the age groups. The highest ICC can be seen in the case of push-ups with a relatively high coefficient of variation (ICC = 0.85, CV% = 54.8%). The extent of variability is the highest in the case of the progressive aerobic cardiorespiratory endurance run (ICC = 0.66, CV% = 61.8%), while standing broad jump (ICC = 0.75, CV% = 23.6) and paced curl-ups (ICC = 0.68, CV% = 31.1) had the lowest variability among the test measures. The standing broad jump also has a relatively high ICC, indicating the higher importance of age on this measure. The lowest ICC could be obtained in the case of the trunk-lift (ICC = 0.48, CV% = 37.7); hence, age has a relatively lower importance on this measure. In the case of the handgrip strength, ICC = 0.66 and CV% = 43.6.

Secondly, a two-block PLS analysis was applied to compare students’ fitness and body composition. The variables of the received data were grouped in two blocks. The first block included physical variables (age, body mass, height, and body fat %), while the performance variables (endurance measuring PACER runs, paced push-ups, standing long jump, etc.) were put in the second block. The variable weights can be seen in Table 2.

The first dimension is responsible for 78.9% of the total explained variance (preserved information), while the second dimension is responsible for 20.6%. Together, the two dimensions preserved 99.5% of the information contained in the dataset, which is excellent.

Blocks represent latent components, and variable weights represent correlation coefficients to these latent components. In the first block, the most influential variables are body weight (r = −0.928; *p* < 0.001) and body fat (r = −0.336; *p* < 0.001). Within the second block, handgrip (r = −0.374; *p* < 0.001) and push-ups (r = 0.276; *p* < 0.001) are the most influential factors. The sign of each correlation coefficient also matters. For example, children with a stronger handgrip had lower scores in push-ups; therefore, handgrip and push-ups negatively correlate with each other because of the opposite signs of their weights. Handgrip decreases its latent component, while a higher score in push-ups increases the latent component. The correlation coefficient between the two different latent components (two blocks) can also be measured. There is a stronger correlation (r = 0.298; *p* < 0.001) between the first and the second block in the first dimension.

Out of the first block variables, body weight and body fat show a positive correlation with handgrip strength (the signs of their weights are the same), which means that a larger body weight results in a stronger handgrip. At the same time, heavier children had lower scores in push-ups and curl-ups.

In the second dimension, a lower but still significant correlation was found between the two blocks (r = 0.176, *p* < 0.001). The second dimension differentiates tall and short children from those short children that have a higher percent of body fat. Tall children with a lower body fat percentage performed relatively better in standing long jumps and paced curl-ups.

Latent scores for dimensions 1 and 2 are calculated as follows:

(4) $$LS_{1}=-0.928\times body\;weight-0.336\times body\;fat+0.843\times \;endurance\;run-0.343\times handgrip+0.276\times \mathrm{push}\text{-}\mathrm{up}+0.268\times \mathrm{back}\text{-}\mathrm{saver}\;\;\mathrm{sit}\text{-}\mathrm{and}\text{-}\mathrm{reach}$$

(5) $$LS_{2}=0.806\times body\;height-0.570\times body\;fat+0.751\times \;standing\;broad\;jump-0.433\times \mathrm{back}\text{-}\mathrm{saver}\;\mathrm{sit}\text{-}\mathrm{and}\text{-}\mathrm{reach}$$

As a consequence, in the first dimension, heavier students with a stronger handgrip and lower scores on push-ups and endurance runs will be situated on the left side of the axis (receives negative scores), while thinner students will be placed on the right side of the axis with higher scores on push-ups and endurance run. In the second dimension, taller students with lower body fat tend to be in the upper part of the axis, with higher scores on the standing broad jump and the back-saver sit-and-reach, and shorter students with lower scores on the standing broad jump and the back-saver sit-and-reach tending to place at the bottom of the axis.

**Figure 1:**
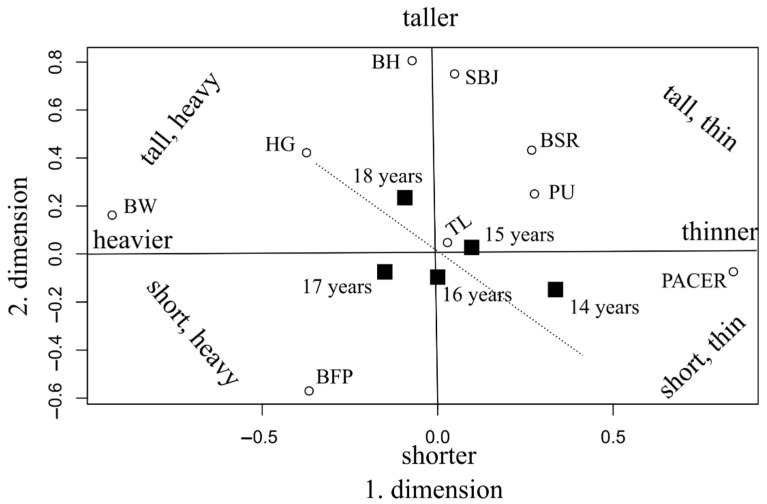
Biplot of the 2-block PLS analysis (age groups are shown as supplementary variables, marked with black squares). BFP: Body fat percentage; BH: Body height; BW: Body weight, BSR: Back-saver sit-and-reach; HG: Handgrip; PACER: Progressive aerobic cardiorespiratory endurance run; PU: Push-ups; SBJ: Standing broad jump; TL: Trunk-lift.

Figure 1 is a graphical representation of the correlations found in the table. In addition, the authors also drew a trend line and presented age groups as a supplementary variable. Standard deviation is normal within the age groups; however, the mean values tend to increase as the age rises. It is 17-year-old students who deviate most from the trend line, as they have a relatively higher body weight and body fat percent, while those aged 14 are relatively thinner.

The figure provides a clear picture of how the two dimensions work by grouping heavier, lighter, taller, and shorter than normal children in separate categories. The trunk-lift can be regarded as an average task, where all of the assessed children could do well, regardless of their age or height, which means that an average child in the population can perform this task. Running in the PACER test was easier for children of average height who were also very thin at the same time, and push-up scores were also higher among children who were relatively shorter and thinner.

The figure shows the variables in a coordinate system where the axes are: thin–fat and tall–short. We see the variables of the surveyed data placed here. For example, considering the ages; it can be seen that at this school, there were even more obese individuals than usual in the 17-year-old age group (Figure 1). This also explains why this age group performed worse in the push-ups at this school, compared to the 15- or 16-year-olds (Table 1). A question is raised as to why the “VO_2_ max” and “Standing long jumps” in this thin–fat and tall–short coordinate system are located where they are in the diagram, since “Standing Broad Jump” is better for tall people (they achieved better results), and obesity does not affect it much. VO_2_ max, the result of which is shown in the figure in the “progressive aerobic cardiorespiratory endurance run” test, works well for thin people, and height does not affect it much.

Students with a short physique seem to have an advantage over taller students in the push-up and trunk-lift tests. Overweight was a definite handicap in both the endurance pendulum and the standing broad jump. Tall-heavy students had a stronger handgrip than students with a thin physique.

Short-thin and tall-thin students performed best in the endurance pendulum, VO_2_ max, back-saver sit-and-reach, and standing broad jump. Tall-thin students had better results in the standing broad jump than short-thin students. Short-thin students performed significantly better than students in other groups on the push-up tests. Short-heavy students did not excel in any of the tests.

## 4. Discussion

The current research confirmed that basic body composition factors such as height, body mass, BMI index, and body fat percentage are major determining factors that highly affect physical fitness test results. Some of the studies evaluating different fitness tests draw attention to the fact that an increase in body mass leads to a decrease in aerobic fitness [25]. The results can only confirm these findings: students with a thin body build were best not only in terms of aerobic fitness performance, but their results proved to be the best in terms of the overall result for all of the fitness tests.

Generally, students with a low body fat percentage had higher scores in endurance shuttle runs, which assess aerobic fitness. The opposite case was also true: having a high body fat percentage resulted in lower performance on the endurance PACER test. A similar conclusion was drawn by an analysis assessing the results of large sample motoric tests conducted on Canadian students. The study revealed that in the past two decades, a slight decrease can be observed in students’ physical fitness due to the increase in body fat percentage and body mass resulting from a sedentary lifestyle and a lack of physical activity [23]. Between 1976 and 2001, a large-sample Finnish study was conducted to examine the change in aerobic fitness in a population aged 13 to 18, which was later supplemented with a questionnaire assessment of BMI and physical activity. This study observed that both boys’ and girls’ fitness performances became worse in the studied period, which the authors considered to result in overweight and obesity becoming more common [24].

Students whose BMI index and body fat percentage were higher than optimal had lower results in the shuttle runs (assessing endurance) and standing long jumps (evaluating muscular strength and endurance). A former Hungarian study also confirms this; the research of Mészáros et al. (2002) [29] conducted in 1975 and then in 2000 showed a decrease in standing long jump results in a sample that comprised young people doing no sports. In 1975, they observed that the 1975 mean of long jumps was 202.75 cm, which decreased to 191 cm by 2000. They considered the increase in body fat per cent to be the reason behind this change, which means they confirmed the spread of obesity not to be favorable in terms of endurance or strength either.

This research’s results showed that tall and heavy students’ handgrip was stronger than those of lean students. Short and heavy students failed to produce outstanding performances on any test; overall, overweight students’ fitness levels were lower than students with optimal body building.

In endurance-assessing shuttle runs, VO_2_ max, paced curl-ups, and standing long jumps, it was the short and lean, and the tall and lean students who had the highest scores, which means that lean students with a low-fat body mass were the most efficient, and that height does not influence students’ performance in these tests.

In terms of the standing long jump, tall and lean students had a better performance than short and lean students; therefore, taller students having longer lower extremities were at an advantage in this test.

In paced push-ups, short and lean students definitely had the highest scores; a significant difference was observed compared to the rest of the groups. The most plausible explanation for these results is that activities requiring extra strength due to the need to carry or move their own weight were disadvantageous for overweight children [42,43].

The overall fitness tests revealed that students with optimal BMI had better results and better fitness levels. This is consistent with the results of a large-sample (*n* = 335,810) Greek study, where students aged 6 to 18 were assessed. Students’ performance in the EUROFITT tests was in negative correlation with obesity, which was evaluated based on the body mass index and abdominal circumference [14].

Overweight and obesity in childhood were identified as health problems in several studies [15,26,44,45,46,47].

In this research, overweight correlated with poorer fitness test results or lower physical fitness level, which is consistent with other studies confirming that overweight or obese children are not as active as they should be—consequently, they are less fit [12,13,14].

The results support that better fitness results in secondary school students can be achieved by improving body build indices, which means that the focus should be on optimal BMI, lowering the body fat percent, and enhancing physical activity.

If we want to improve fitness, we must first focus on enhancing cardiorespiratory endurance; its dominant importance in fitness has been highlighted in several studies. In addition, plenty of studies draw attention to the fact that sports and physical activity play an important role in health improvement and prevention, both in children and adults [48,49,50,51,52].

As several studies have observed, an active lifestyle and decreasing inactivity positively correlate with general fitness level; all of these studies promote an active lifestyle and doing sports [53]. In Hungary, the introduction of daily P.E. classes (for students aged 6 to 18) resulted in an increase in students’ physical activity. However, this is not enough; even more promotion of sports for children is suggested to help them be healthier adults than their parents are. Unfortunately, although Hungarian adults are highly active at their workplace and in housework, they are still the fourth in the world in terms of obesity, due to a lack of physical leisure activities and sports activities [54].

## 5. Conclusions

The major conclusion of this research is that overweight is in negative correlation with the examined elements of fitness tests; also, this is the most highly determining factor of low fitness levels.

Physical fitness test results were determined in the largest part by basic physical characteristics such as height, weight, BMI index, and body fat percentage. Looking at the composite scores of the NETFIT^®^ test, it is apparent that students with a thin physique had the best results. In the endurance pendulum, students with low body fat percentages faired better than their peers with higher body fat percentages, who had rather poor results.

In order to compensate for the widespread sedentary lifestyle among students, to increase physical activity, to reduce obesity, and to increase fitness, we recommend that the school intervene in two areas: nutrition and programs that encourage sports and exercise.

Reforming the diets of students can help create an optimal BMI and reduce the body fat percentage. The canteen in the school could be reformed so that the intake of vegetables and fruits could increase. By organizing health weeks, information could even be given to students in a playful form, and could end with a competition. By organizing reform kitchen cooking afternoons, students can be motivated to prepare healthy meals and to acquire practical knowledge related to this. To encourage physical activity and sports, students could be offered more school sports opportunities. Competition between classes could be organized, using ICT tools and applications. Various challenges and accomplishments could be announced to win the title of the healthiest class, where we could encourage them to change their lifestyles by offering prizes and smaller prizes to the winners.

This was the first study in Hungary in which the data of an entire school was processed, and the NETFIT^®^ results and body composition indicators were examined. Its novelty lies in the fact that, focusing on the age group of young boys (14–18) (except for those who were excused due to illness and health problems), a survey on almost all the students from a vocational training center was conducted and analyzed. Then, it was compared to the national data, which laid the foundation for the school’s local areas of intervention, in order to formulate a recommendation plan that helps to create a healthier and fitter society and employee class. At the same time, two proposed areas of intervention were included in the research to compensate for the widespread sedentary lifestyle among students, to increase physical activity, to reduce obesity, and to increase fitness (one is the area of nutrition, and the other is programs that encourage sports and exercise).

## 6. Limitation

The main limitation of our study is that it examined only male students in Budapest, the reason for this being the fact that due to the training scheme, only boys attend the Technical Training Centre. The sample includes many students from the country and living in dormitories, which makes the socioeconomic background variables inhomogeneous. A limitation of the present study is the biological maturity of the sample. We did not measure the peak height velocities of the children.

## Figures and Tables

**Table 1:** Basic statistics of the NETFIT^®^ performance variables showing significant differences.

	Age Group	National Data		School Data		*t*-Statistics
		Mean	Standard Deviation	Mean	Standard Deviation	
Paced push-ups (number)	14	17.4	9.7	26.5	10.1	5.49 **
	15	19.0	9.5	30.6	19.1	9.22 **
	16	20.2	9.8	29.3	12.8	9.49 **
	17	20.9	10.1	27.2	13.9	5.56 **
	18+	21.3	10.3	36.7	20.0	7.27 **
VO_2_ max (mL/kg/min)	15	46.2	8.1	36.4	19.7	−7.23 **
	16	45.5	8.2	40.4	21.1	−2.96 **
	17	44.5	8.2	35.9	23.7	−4.24 **
Paced curl-ups (number)	15	59.5	23.5	70.0	18.6	8.78 **
	16	59.7	23.5	67.0	20.3	4.97 **
	17	59.8	23.4	66.6	22.7	3.76 **
	18+	58.7	23.7	64.5	23.0	2.38 **
Handgrip strength (kg)	14	35.5	9.1	39.9	10.0	2.83 **
	17	45.9	9.5	40.4	14.2	−4.94 **
Standing long jumps (cm)	15	194.0	33.0	184.4	46.5	−3.18 **
	16	200.3	32.6	189.0	41.2	−3.70 **
	17	204.0	33.3	189.1	45.5	−4.26 **

**: *p* < 0.01.

**Table 2:** Two-block PLS dimensions and their relation to the variables of the two blocks.

Matrix	Variable	Dimensions	
		1 (Thinner)	2 (Taller)
F1 (Block1)	body height (BH)	−0.073	0.806
	body weight (BW)	−0.928	0.162
	% of body fat (BFP)	−0.336	−0.570
F2 (Block2)	progressive aerobic cardiorespiratory endurance run (PACER)	0.843	−0.074
	trunk-lift (TL)	0.016	−0.058
	standing broad jump (SBJ)	0.048	0.751
	handgrip (HG)	−0.374	0.422
	push-ups (PU)	0.276	0.250
	back-saver sit-and-reach (BSR)	0.268	0.433
Correlation		0.298 *	0.176 *
Variance explained		78.9%	20.6%

*: both coefficients are significant at 0.1 level as *p* < 0.001.

## Data Availability

Not applicable.
